# Supplementary material for: Galectin-9 interacts with Vamp-3 to regulate cytokine secretion in dendritic cells
Source: Cell Mol Life Sci. 2023 Sep 27;80(10):306. doi: 10.1007/s00018-023-04954-x (PMC10533640; doi:10.1007/s00018-023-04954-x)
Supplement: Supplementary file 1 — Supplementary file1 (PDF 19868 KB) [file 18_2023_4954_MOESM1_ESM.pdf]

Supplementary Table 1. Primer sequences

Gene name	Forward primer	Reverse primer
hIL12A	CACAAAAGATAAAACCAGCA	CTCTCTGGAATTTAGGCAAC
hIL6	GACAGCCACTCACCTCTTCAGAACG	ATCCATCTTTTTCAGCCATCTTTGG
hIL10	TCAAGGCGCATGTGAACTCC	GATGTCAAACTCACTCATGGCT
hTNFα	ATGAGCACTGAAAGCATGATCC	GAGGGCTGATTAGAGAGAGGTC
ACTB	CTGGAACGGTGAAGGTGACA	AAGGGACTTCCTGTAACAACGCA

Supplementary Figure 1

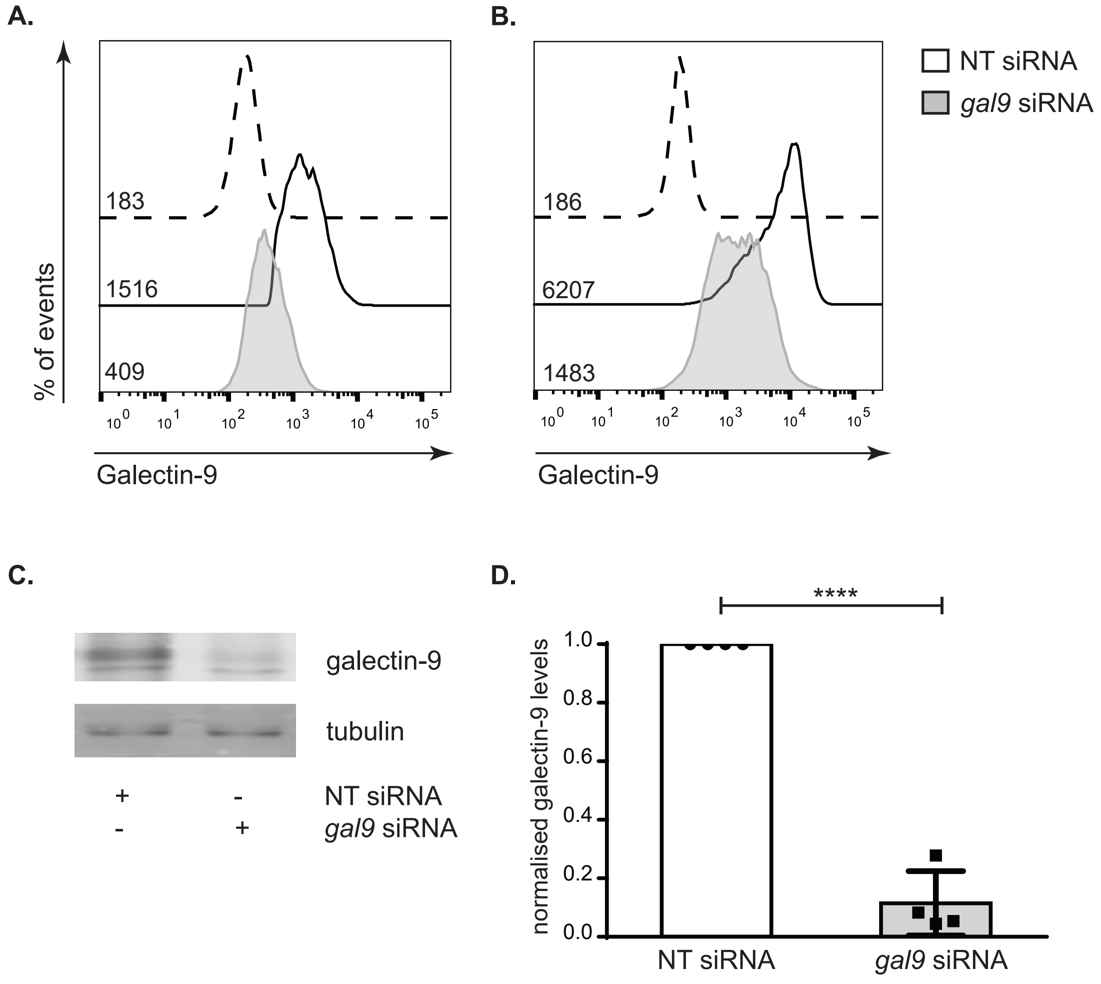

Supplementary figure 1. Endogenous levels of galectin-9 in moDCs can be modulated. Day 3 moDCs were transfected with gal9 siRNA or a NT siRNA. Levels of galectin-9 were assessed by flow cytometry 48 h after transfection. **A**. Graph shows galectin-9 levels at the plasma membrane. **B.** Histogram depicts total protein levels, both surface-bound and intracellularly. As shown, galectin-9 was depleted both at the plasma membrane as well as intracellularly. NT (black line, unfilled population), gal9 siRNA-transfected moDCs (light grey population). Black dotted line represents isotype control values. Graphs are representative for one donor and numbers in inset indicate geometric mean fluorescence intensity (gMFI). **C.** Total lysates from NT and gal9 siRNA transfected cells were subjected to Western Blot and Galectin-9 expression was analysed. Tubulin was used as loading control. Immunoblot is representative of four independent experiments. D. Graph depicts galectin-9 levels in galectin-9 depleted moDCs normalised to tubulin and made relative to the corresponding NT siRNA sample. Graph shows mean ± SEM of four independent donors. **** $p < 0.0001$.

Supplementary Figure 2

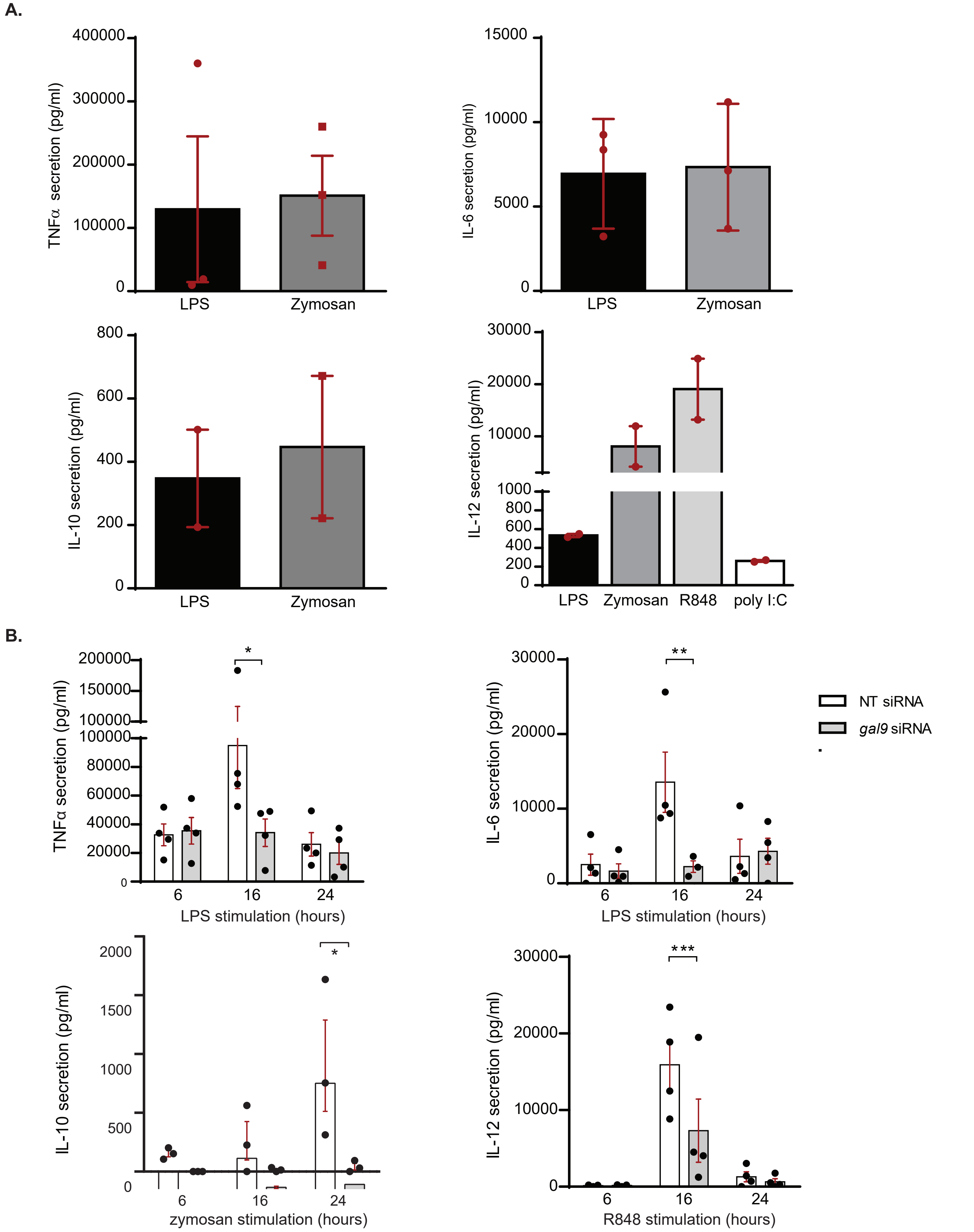

Supplementary Figure 2. Several stimuli enhance the secretion of specific cytokines. A. NT siRNA transfected moDCs were seeded on day 6 and treated with either 1 µg/ml LPS, 4 µg/ml R848, 1 µg/ml poly I:C or Zymosan particles (ratio 1:10) for 16 prior to supernatants being collected. Specific ELISA reactions for the above cytokines were performed. Graphs show mean ± SEM of two or three independent donors. **B.** Day 3 moDCs were transfected with NT siRNA or gal9 siRNA. Forty-eight hours after transfection, cells were treated with LPS, R848 or zymosan particles for the indicated time points and secreted levels of TNFα, IL-6, IL-10 and IL-12 determined by ELISA. Graphs show mean ± SD of four independent donors. Two-way ANOVA was conducted between NT and gal9 siRNA samples. * $p < 0.05$; ** $p < 0.005$; *** $p < 0.001$.

Supplementary Figure 3.

A.

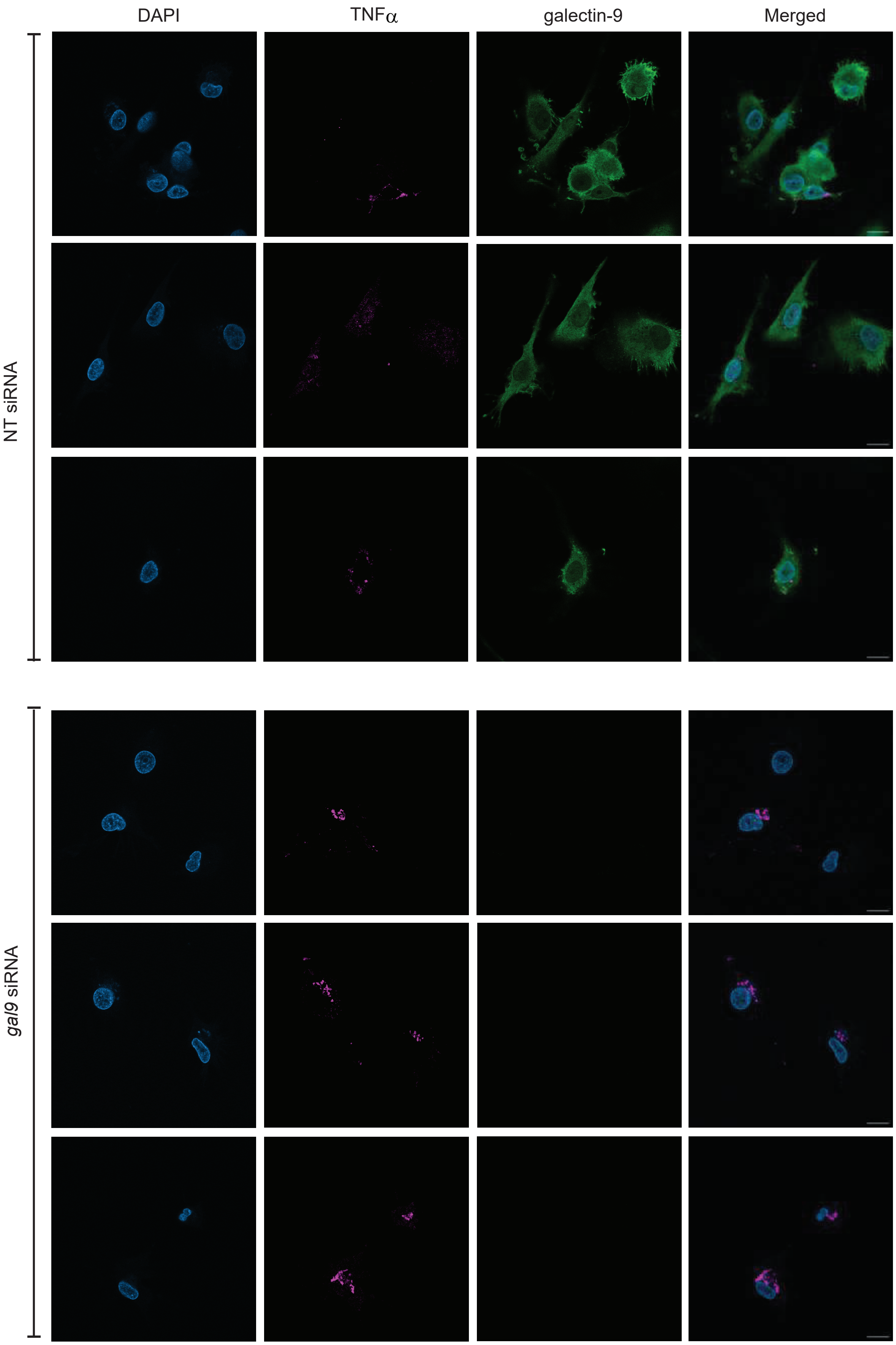

Supplementary Figure 3. Cytokines accumulate intracellularly upon galectin-9 depletion. A. Additional representative images of the results shown in Figure 2C. DAPI, TNFα and galectin-9 immunofluorescence stainings from NT (above) and gal9 siRNA (below) transfected moDCs treated for 6 hours with 1 μg/ml LPS are shown. Scale bar: 10 μm.

Supplementary Figure 4

A. **B.**

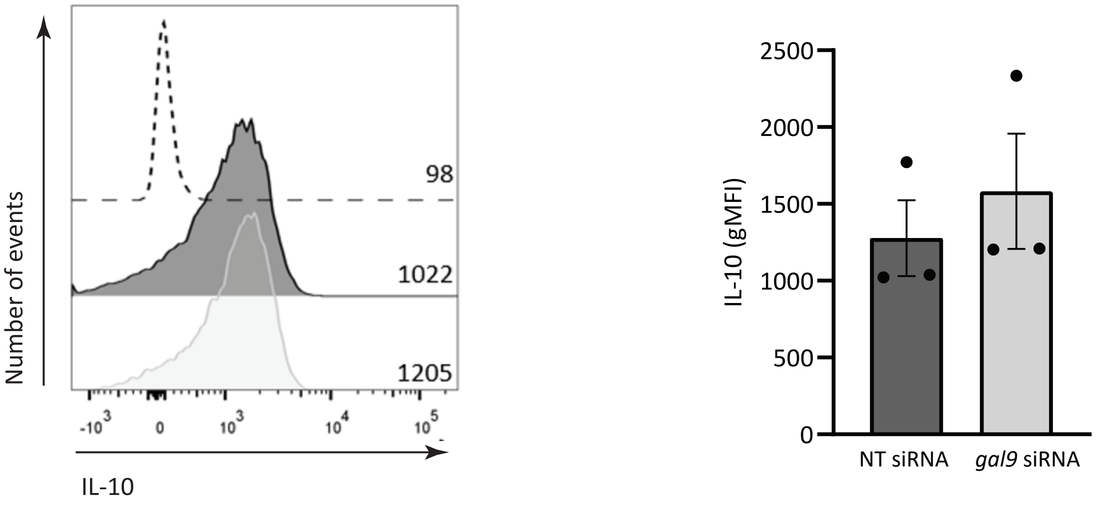

Supplementary Figure 4. Cytokines accumulate intracellularly upon galectin-9 depletion. A. Day 5 NT and gal9 siRNA transfected moDCs were treated for 6 hours with Zymosan (ratio 1:10) prior to being harvested and the intracellular levels of IL-10 analysed by flow cytometry. Dotted line shows TNFα levels in untreated cells. Dark grey depicts NT siRNA DCs and light grey galectin-9 depleted DCs (gal9 siRNA). Numbers represent geometric mean fluorescent intensity. **B.** Quantification of results shown in (A) for three independent donors.

Supplementary Figure 5

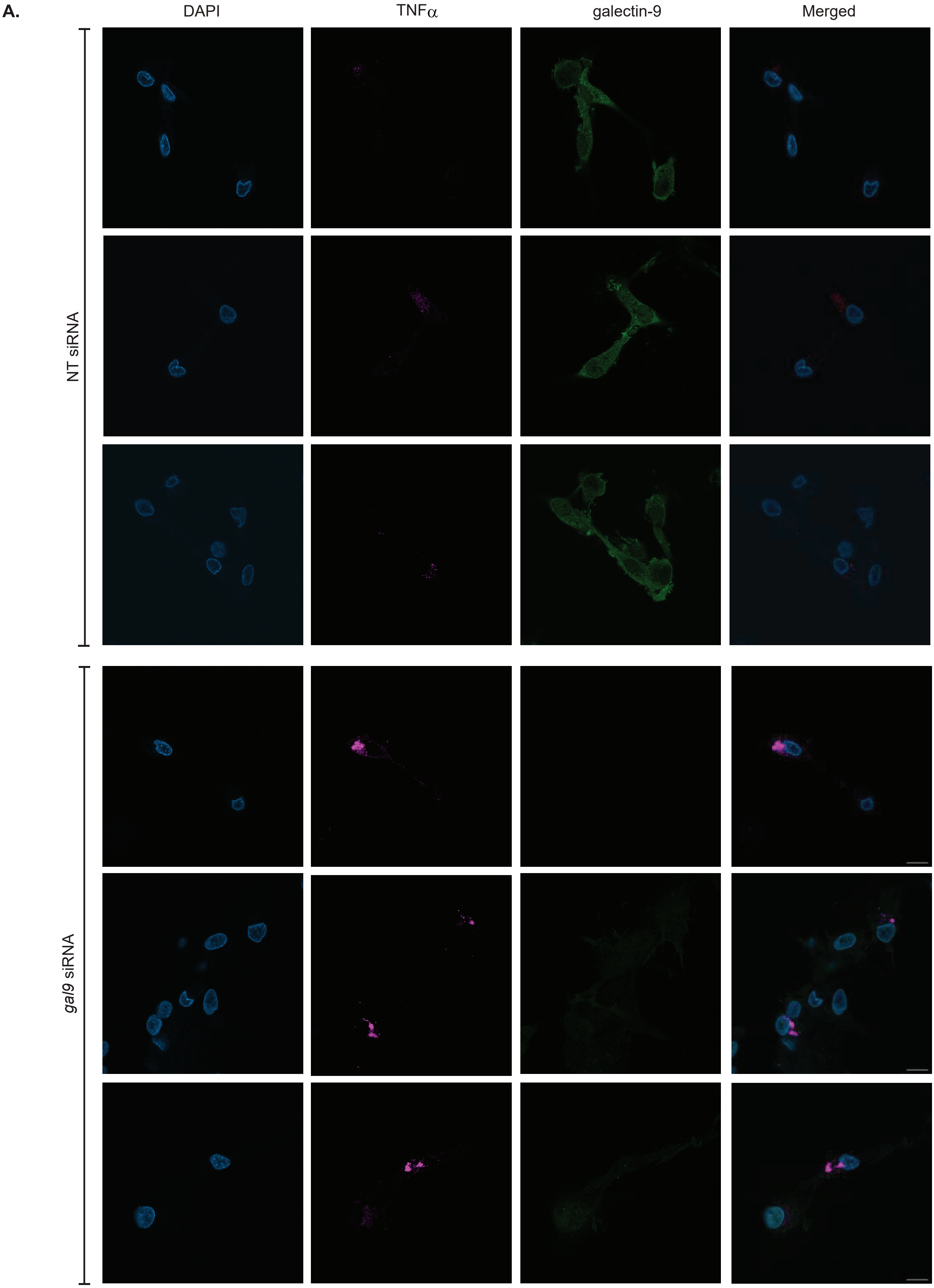

Supplementary Figure 5. Galectin-9 depleted moDCs eliminate intracellular cytokines via lysosomes. Additional representative images of the results shown in Figure 3C. DAPI, TNFα and galectin-9 immunofluorescence stainings from NT (above) and gal9 siRNA (below) transfected moDCs treated for 6 h with 1 µg/ml LPS and Bafilomycin (200 µM) are shown. Scale bar: 10 µm. For clarity purposes, the merged image in the NT moDCs has been generated without the galectin-9 staining and only including the signal for DAPI and TNFα.

Supplementary Figure 6.

A.

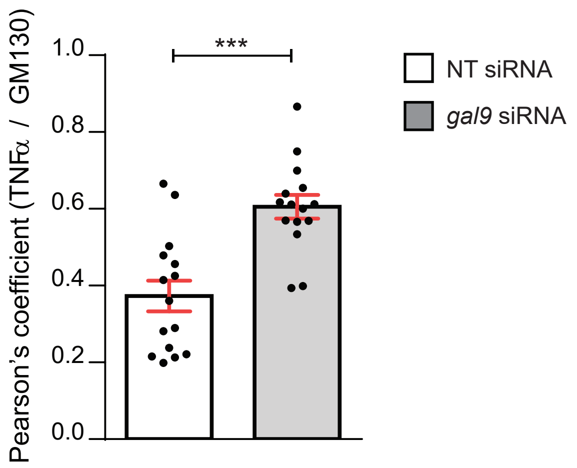

B.

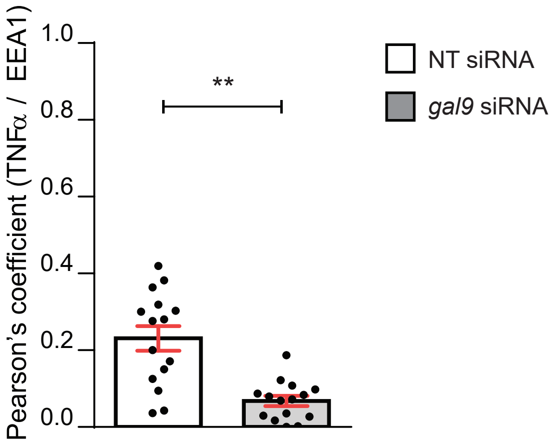

C.

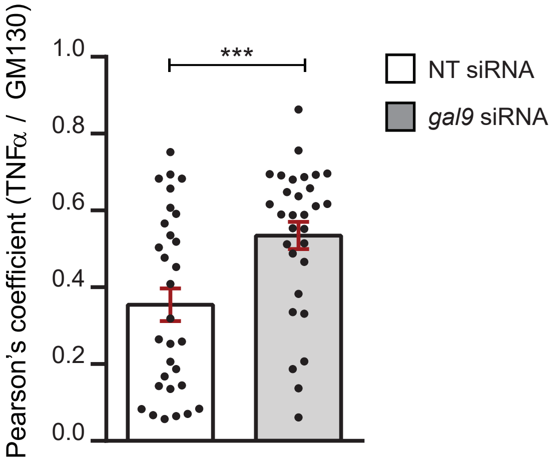

Supplementary Figure 6. Supplementary Figure 6. Pearson's correlation coefficients. A. NT and gal9 siRNA-transfected moDCs were treated with 1 µg/ml LPS for 2 hours after which cells were fixed and stained with specific antibodies against TNFα, the Golgi marker GM130. Graph shows Pearson's correlation coefficient of cells shown in main Figure 4B. Fifteen cells from two representative donors are quantified. **B.** NT and gal9 siRNA- transfected moDCs were treated with 1 µg/ml LPS for 6 hours after which cells were fixed and stained with specific antibodies against TNFα, EEA1. Graph shows Pearson's correlation coefficient of cells shown in main Figure 4C. Fifteen cells from two representative donors are quantified. **C.** NT and gal9 siRNA-transfected moDCs were stained with specific antibodies against the Golgi marker GM130. Graph depicts Pearson's correlation coefficient for images shown in main Figure 5D. For all panels data represent mean correlation coefficient ± SEM and paired student's t-test was conducted between NT and gal9 siRNA transfected moDCs. ** $p < 0.005$; *** $p < 0.001$.

Supplementary Figure 7.

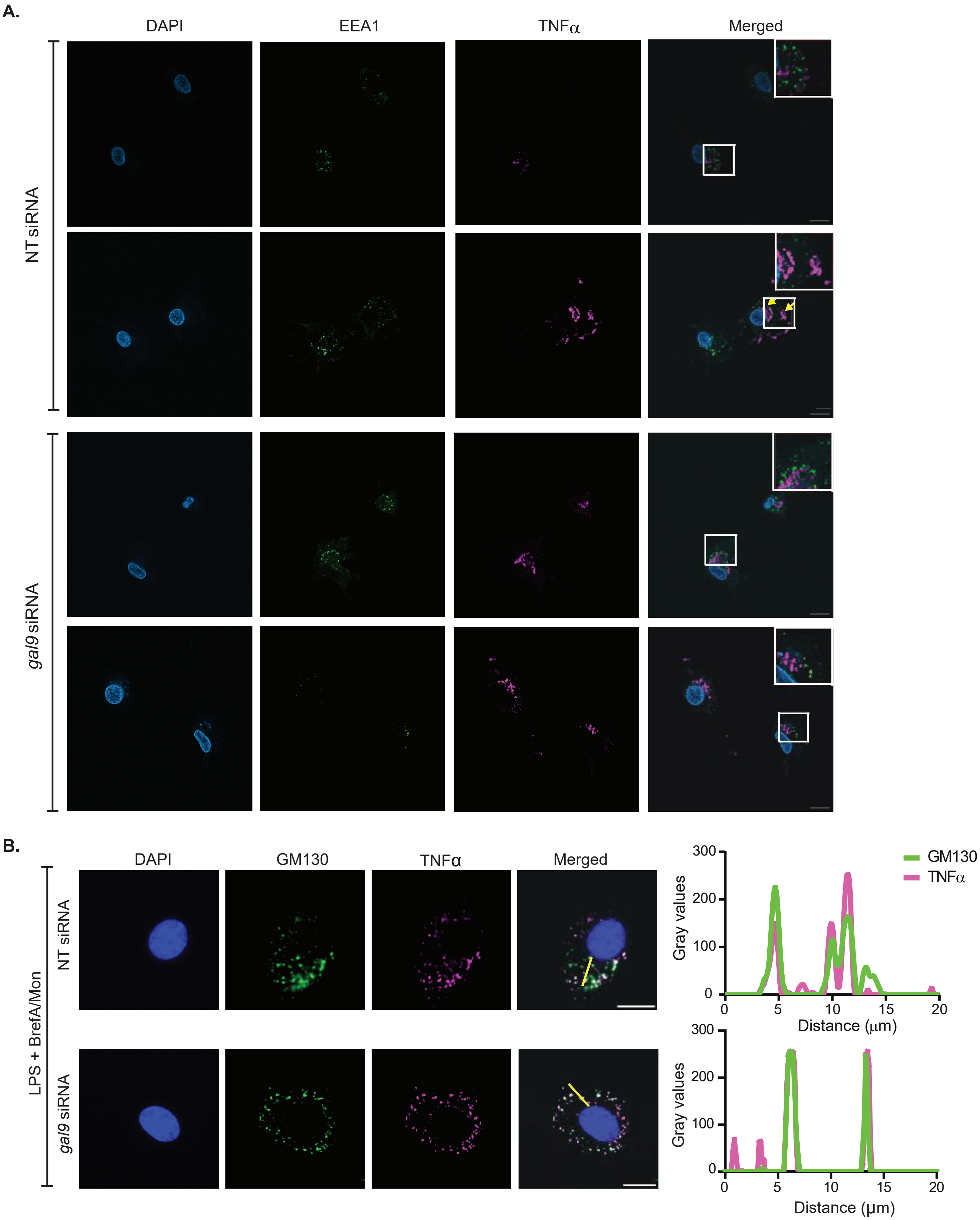

Supplementary Figure 7. Cytokines do not traffic after the Golgi complex in galectin-9 depleted moDCs. A. Additional representative images of the results shown in Figure 4C. DAPI, TNFα and EEA1 immunofluorescence stainings from NT (above) and gal9 siRNA (below) transfected moDCs treated for 6 hours with 1 μg/ml LPS are shown. Arrows indicate sites of colocalisation between TNFα and EEA1. **B.** NT and gal9 siRNA moDCs were treated with 1 μg/ml LPS in combination with the Golgi inhibitors Brefeldin A (BrefA, 10 μg/ml) and Monensin (Mon, 2 μM) after which cells were fixed and stained with specific antibodies against TNFα, the Golgi marker GM130 and DAPI for nuclear staining. Representative airyscan confocal images of three independent experiments are shown. Scale bar: 10 μm. Graphs: fluorescent cross-sections as indicated.
